# Primary Spinal Intradural Mesenchymal Chondrosarcoma with Several Local Regrowths Treated with Osteoplastic Laminotomies: A Case Report

**DOI:** 10.1055/s-0037-1604159

**Published:** 2017-07-20

**Authors:** Marek Derenda, Damian Borof, Ireneusz Kowalina, Wojciech Wesołowski, Wojciech Kloc, Ewa Iżycka-Świeszewska

**Affiliations:** 1 Department of Neurosurgery, Regional Hospital, Elblag, Poland; 2 Department of Pathology, Regional Hospital, Elblag, Poland; 3 Departments of Neurology & Neurosurgery, University of Varmia & Masuria University, Olsztyn, Poland; 4 Departments of Pathology & Neuropathology, Medical University of Gdansk, Poland

**Keywords:** mesenchymal chondrosarcoma, intradural tumor, spinal tumor, neurosurgery, adjuvant treatment

## Abstract

Mesenchymal chondrosarcomas (MCSs) are rare malignant tumors of the bone and soft tissues. Only a few cases of such tumors originating from the spinal canal meninges have been described in the literature. The authors report on a case of a 22-year-old woman with MCS of the arachnoid at the T12-L1 level with a 14-year-long observation. The tumor was totally resected using osteoplastic laminotomy with reconstruction of laminar roof. This small spindle cell tumor was initially microscopically suspected of synovial sarcoma, but correctly verified with widened immunophenotyping and molecular studies as MCS. At its first recurrence, the neoplasm showed microscopically a typical bimorphic pattern of small round cell component with foci of hyaline cartilage. The patient experienced three local recurrences: 4, 6, and 10 years after the initial resection, respectively. The techniques of laminotomy and relaminotomy were also used during three following operations. The repeated surgical removal, radiotherapy, and chemotherapy were the methods of complex oncological treatment. The patient remains now in complete remission, fully self-dependent with slight motor disturbance, and mild sensory deficits. Current views on the clinicopathological characteristics and treatment modalities of the chondrosarcomas of the spinal canal are discussed.

Chondrosarcomas of the spine constitute 4 to 10% of all primary bone tumors. Although the majority of them originate in bone, some of the tumors have an extraosseous origin. 1 Mesenchymal chondrosarcoma (MCS) constitutes only up to 10% of all primary chondrosarcomas, including approximately one-fourth of cases involving soft tissues. Histological differential diagnosis of MCS can be difficult and includes dedifferentiated chondrosarcoma, fibrosarcoma, small cell osteosarcoma, synovial sarcoma, and Ewing's sarcoma/primitive neuroectodermal tumor group of tumors. Extraskeletal MCS relatively frequently develop in association with the meninges, mainly intracranially, much more rare in the spinal region. Intradural, extramedullary location without dural attachment of the tumors is extremely rare. MCS are characterized by a protracted outcome with local recurrences. 2 3 4 5 6 7 8

## Case Report

**Fig. 1:**
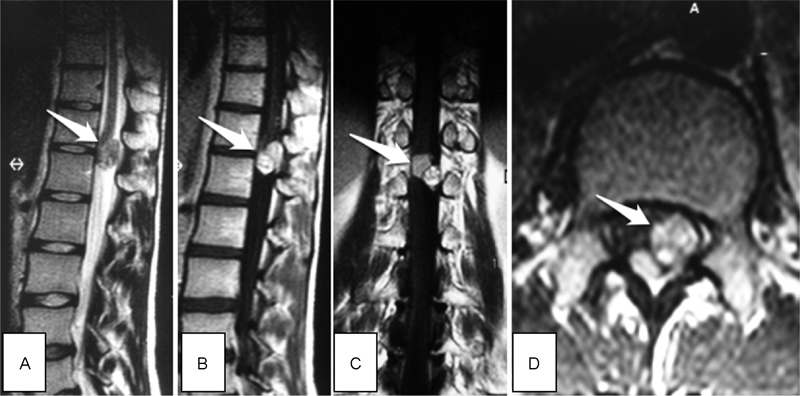
First manifestations of the tumor in 2002 in MR imaging (the arrow points to the tumor). ( **A** ) Preoperative sagittal T2. ( **B** ) Preoperative sagittal T1 + C. ( **C** ) Preoperative coronal T1 + C. ( **D** ) Preoperative axial T1 + C. MR, magnetic resonance.

A 22-year-old woman was admitted to our hospital's outpatient department in 2002 with 2 months history of bladder and bowel incontinence, intermittent pain in the left hip and groin region radiated to the knee, which increased in standing and sitting position. Moreover, she complained of some “pins and needles” sensations in buttocks, thighs, and right great toe regions. Physical and neurological examinations showed hypoesthesia below the dermatome L1 on the left side, absence of the deep tendon reflex in knee and ankle, positive Laseque's sign, and femoral nerve stretch test in both sides. Spinal magnetic resonance (MR) imaging revealed an intradural, extramedullary mass measuring 22 × 19 × 12 mm at the T12-L1 level on the left side of the spinal cord. The more caudally located part of the tumor appeared hyperintense on T1- and T2-weighted images. The more rostrally located part of the tumor appeared isointense on T1- and T2-weighted images, but very strong enhancement of this part of the tumor was observed after intravenous gadolinium administration ( Fig. 1 ).

The tumor was totally resected via osteoplastic laminotomy of T12-L1 with reconstruction of the spinal canal roof. Reconstructive technique was used to avoid spine deformations and to achieve a good cosmetic result. A bluish, well-vascularized soft mass was found subdurally and subarachnoideally with a single trophic artery. It was possible to slide the arachnoid that covered the tumor down from its surface. The second part of the tumor was hard, white-yellowish, unvascularized mass, closely adhering, and focally originating from the arachnoid. Using microsurgical technique, the tumor was removed completely. After the operation, the neurologic status improved gradually, and symptoms regressed.

Histopathological examination revealed hypercellular mesenchymal neoplasm composed of undifferentiated small round and spindle-shaped cells. Immunophenotype showed vimentin, EMA, CD99, and bcl2 positivity, together with S100, desmin, and cytokeratin negativity of the neoplastic cells. Proliferation index Ki-67 was 2%. Preliminary diagnosis was synovial sarcoma. Further studies with fluorescence in situ hybridization confirmed MCS by the exclusion of translocation typical for Ewing's sarcoma t(11,22) and by exclusion of translocation t(X,18) typical for synovial sarcoma.

**Fig. 2:**
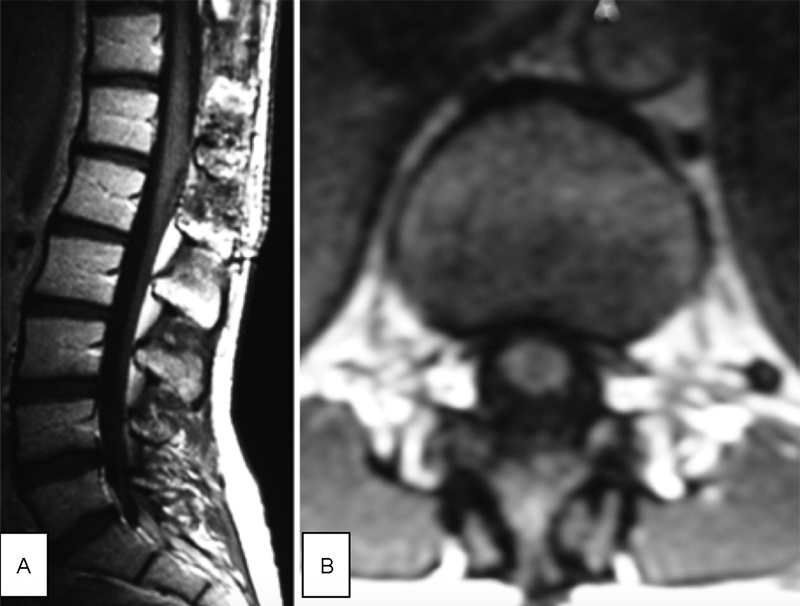
Postoperative control MR imaging performed 6 months after operation showed no evidence of the tumor. ( **A** ) Sagittal T1 + C. ( **B** ) Axial T1 + C. MR, magnetic resonance.

Eight weeks after surgery, the patient received local irradiation in total dose of 4,980 cGy in 28 fractions during 37 days of radiotherapy. Four months after the operation, the patient was free from symptoms, with persistent hyporeflexia of the deep tendon in the left knee and ankle. Several MR images performed during 3 years after surgery did not show any residual or recurring intraspinal mass ( Fig. 2 ). The patient's general condition was very good. She resumed work as a shop assistant; 2.5 years after the surgery, she gave birth to her first child.

**Fig. 3:**
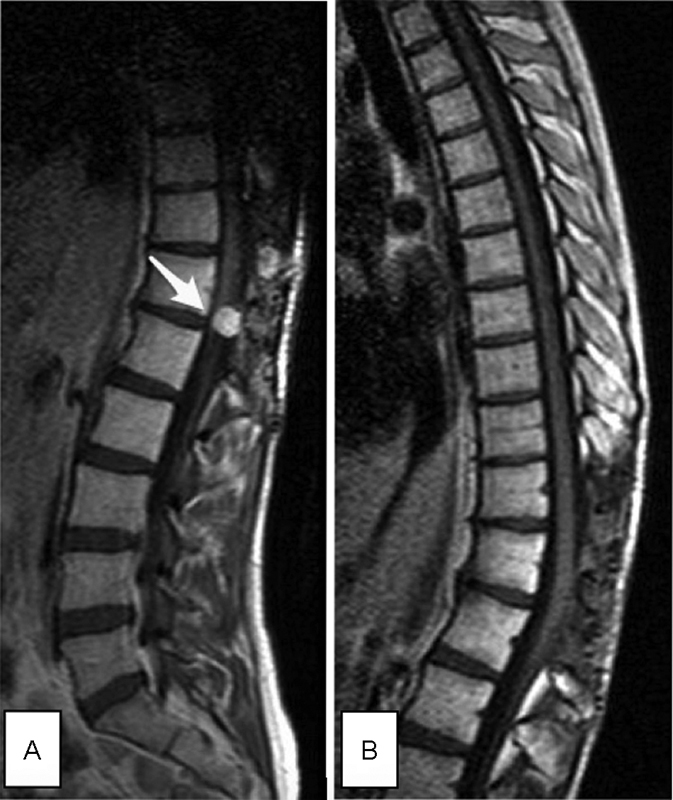
First recurrence of the tumor in 2006 in MR imaging. ( **A** ) Preoperative sagittal T1 + C (the arrow points to the tumor). ( **B** ) Postoperative control MR imaging performed 6 months after operation—sagittal T1 + C—showed no evidence of the tumor. MR, magnetic resonance.

**Fig. 4:**
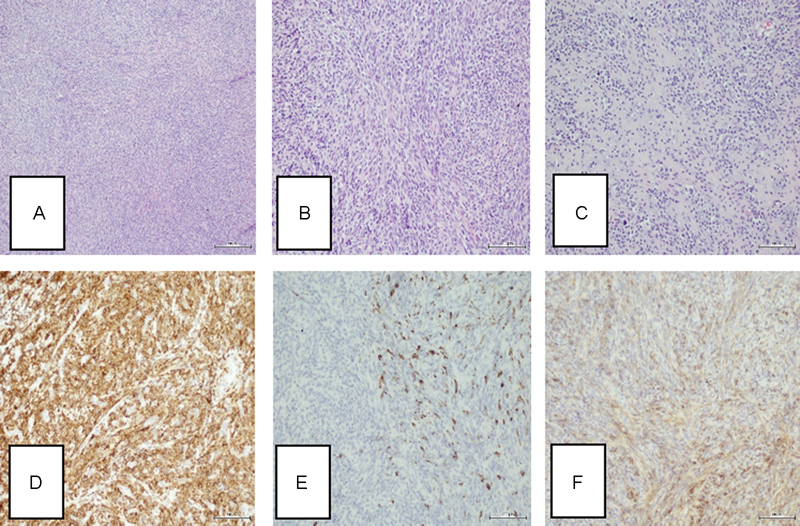
Histopathological features of tumor. ( **A** ) The main component of tumor made of monotonous small round blue cells (HE ×50). ( **B** ) Small, malignant cells with low nuclear pleomorphism and atypia (HE ×100). ( **C** ) The second component of tumor—well-differentiated cartilage areas (HE ×100). ( **D** ) Diffuse and strong Bcl-2 immunostaining (bcl-2, ×100). ( **E** ) Scattered cells positive for desmin (desmin, ×100). ( **F** ) Diffuse membranous CD99 immunoreactivity (CD99, ×100). HE, hematoxylin and eosin.

In 2006, 4 years after the initial treatment, control MR imaging revealed an intradural, extramedullary Th12-L1 level mass, measuring 15 × 15 × 15 mm. The mass was isointense on T1-, T2-weighted images and strongly homogenously contrast enhancing after intravenous gadolinium administration ( Fig. 3A ). Relaminotomy of Th12-L1 was performed and the tumor was totally resected using microsurgical technique. Histologically, the resected tumor revealed biphasic pattern of MCS. Poorly differentiated, malignant component made of small round-to-oval blue small cells was predominant ( Fig. 4A, B ). This pattern was intermixed with comparatively scant hyaline cartilage areas—the second, less conspicuous, component of the tumor. Areas of cartilage were well differentiated with discrete cellular and nuclear atypia ( Fig. 4C ). Sheaths of closely packed small cells were arranged around staghorn-shaped vessels. Neoplastic cells showed strong positive reaction with bcl-2 antibody ( Fig. 4D ), while S-100 protein was positive in cartilage areas. Desmin decorated some scattered small cells ( Fig. 4E ) and CD99 showed moderate membrane reactivity ( Fig. 4F ). Epithelial membrane antigen, cytokeratin AE1/3 cocktail, neuron specific enolase (NSE), and synaptophysin were negative. Proliferation Ki-67 index was low, ∼2%.

Postoperative neurologic condition of the patient was good. Fourteen weeks after the surgery, the patient was treated with three cycles of chemotherapy (vincristine, doxorubicin,cyclophosphamide [VadriaC], etoposide, ifosfamide, and dactinomycin). The tolerance of chemotherapy was generally poor. The last cycle had to be modified and it was performed without etoposide, ifosfamide, and dactinomycin.

**Fig. 5:**
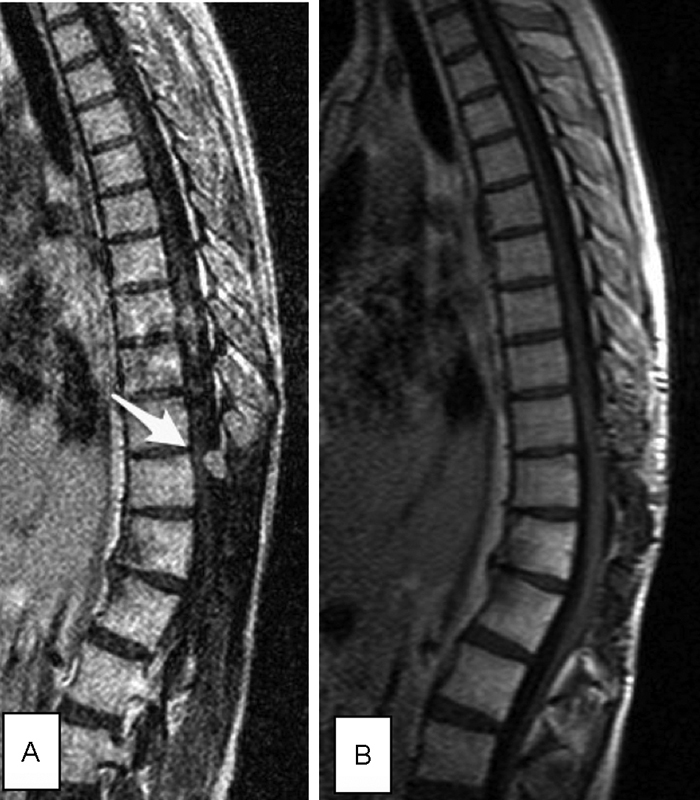
Second recurrence of the tumor in 2008 in MR imaging. ( **A** ) Preoperative sagittal T1 + C (the arrow points to the tumor). ( **B** ) Postoperative control MR imaging performed 6 months after operation—sagittal T1 + C—showed no evidence of the tumor. MR, magnetic resonance.

Since 2008, the patient began to complain of persistent local pain in the thoracolumbar region. Neurologic examination revealed decrease of muscle power of flexors and extensors of the left foot. MR imaging showed at the T10-T11 level 11 × 8 × 13 mm extradural mass, hyperintense on T1-weighted and short tau inversion recovery images, hypointense on T2-weighted and proton density images, and contrast enhancing ( Fig. 5A ). Uncomplicated osteoplastic laminotomy of T11 and relaminotomy of T12 were performed and tumor was resected. After this intervention, the neurologic condition of the patient was stable for 5 years.

**Fig. 6:**
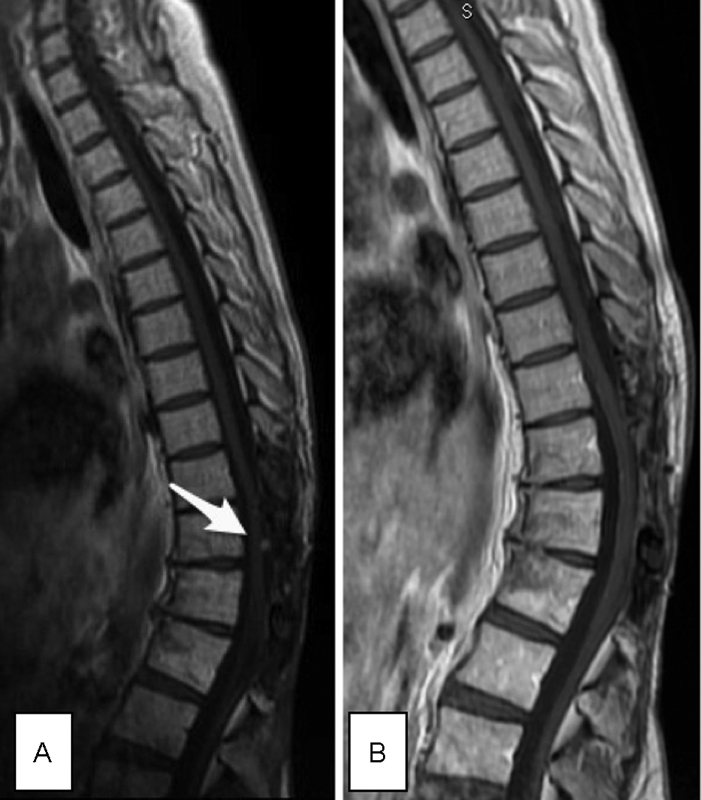
Third recurrence of the tumor in 2014 in MR imaging. ( **A** ) Preoperative sagittal T1 + C (the arrow points to the tumor). ( **B** ) Postoperative control MR imaging performed 32 months after operation—sagittal T1 + C—showed no evidence of the tumor. MR, magnetic resonance.

In 2013, routine control MR imaging detected subdural mass measuring 2 × 1 mm on posterior surface of the spinal cord at the T11 level, at the same place as in 2008. The tumor increased to 3 × 4 × 5 mm 3 months later ( Fig. 6A ). The fourth operation with osteoplastic relaminotomy of T11-T12 was performed. Histology and proliferation rate of the lesion were same as earlier.

In 2016 (32 months after last neurosurgical intervention), there is no evidence of local recurrence or metastatic mass in control MR imaging ( Fig. 6B ). The patient is fully self-dependent, although she complains of periodically recurrent mild back pain in the thoracolumbar area and tactile hyperesthesia in the region of left crus and foot. Decreased strength of the extensors of the left foot is still persistent.

Several MR images performed during 14 years of follow-up revealed gradually increasing kyphotic deformation of the spine at the T11-L1 levels. The patient was informed about the necessity for stabilization, but rejected this option justifying her decision by minor afflictions at the moment. Adjournment of the fixation of the spine is a result of one more reason. The absence of extensive titanium implants allows for avoiding disturbances of imaging of neural structures. Hence, it was possible to detect even very small recurrences of the tumor, starting from a few millimeters in size. However, there is no doubt that the fixation of the kyphotic segment will be needed.

## Discussion

In 1959, Lightenstein and Bernstein introduced the term “MCS” describing two cases of primitive chondrosarcoma of the bone, showing distinctive histology. 2 In 1964, Dowling described the first such case confirmed to be of nonosseous origin based on autopsy. 9 MCS accounts for ∼10% of all chondrosarcomas 10 and 0.25% of bone neoplasms. 11 Approximately 70% of the cases occur during the second and the third decades of life. 3 This malignant neoplasm is thought to derive from primitive cartilage-forming mesenchymal tissue. It is characterized by the presence of solid, highly cellular areas composed of round or slightly spindled primitive mesenchymal cells with foci of cartilaginous differentiation. 3 Although a majority of these tumors are believed to arise from bone, there is a considerable percentage ranging from 33 to 50% that originates in the extraskeletal soft tissue and then most often involving the meninges. 10 11 Schneiderman et al suggested that extraskeletal MCSs are more common than previously reported, reaching 60%, based on analysis of numerous Surveillance, Epidemiology, and End Results database. 12 Most of the tumors arising from meninges were reported to be intracranial in location and very rarely within the spinal canal. 3 4 5 6 9 10 11 13 14 15 16 17 18 19 20 21 22 23 24 25 26 27 28 In Harsh and Wilson review concerning 16 MCS of the primary central nervous system, only five occurred in intraspinal region. 18 These tumors have been found more frequently in an extradural location, with the majority having a dural attachment. Forbes and Eljamel in literature review of 31 meningeal chondrosarcomas reported 11 spinal ones. 25 In 2012, Obuchowicz et al reported 24 cases of intraspinal MCS in children, adolescents, and young adults as described in literature until 2010. 26 Intradurally located MCSs are described incidentally, thus Andersson et al found 15 intraspinal (meningeal) such cases until 2014. 27 A few publications described primary intraspinal dumbbell-shaped MCS. 15 16 19 20 28 29 30 In 2014, Lee et al reported a case of multiple intradural extramedullary masses of MCS at C7 to L5 spinal levels. 31 Kotil et al reported an intradural, but myxoid chondrosarcoma, arisen from pia mater at the T12 level. 32 Li and Yao reported spinal intradural MCS arising from the pia mater at the T11-L1 level in a 3-year-old girl. 6 We report almost identical case of intradural tumor, originating from the arachnoid. The exact histogenesis of intradural chondrosarcomas is obscure because these lesions are usually associated with cartilage. 20 It seems possible that chondrosarcomas could arise from the dura because it has this periosteal component over the spinal extradural vault. These chondrosarcomas may arise from embryonic rest cells of cartilage within the dura. 28 The most probable hypothesis proposes its origin from primitive multipotential mesenchymal cells. 33

Histologically, most MCSs present biphasic pattern of foci of chondroid differentiation and poorly differentiated mesenchymal component. Some tumors have cartilaginous areas evident in very small samples, in the others, small mesenchymal cells predominate. Chondroid areas can be sharply demarcated or blend gradually with undifferentiated areas. The small round cell component is similar to Ewing's sarcoma or spindle-monophasic synovial sarcoma (SS) with hemangiopericytoma-like vascular pattern. Histological features do not predict patients' prognosis. Proliferative and mitotic index is usually low. Immunophenotype is not very specific: CD99 and bcl-2 are usually positive among small cell component, vimentin is usually diffusely positive in both, and S100 marks chondroid component. Desmin and actin decorate single small cells, while EMA, cytokeratins, and neuroendocrine markers are usually negative. Synovial sarcoma and Ewing's sarcoma create the biggest differential diagnostic problem to be resolved by immunophenotyping. Sometimes molecular studies are necessary to exclude aberrations typical for above-mentioned sarcomas such as t(X,18) and t(11,22). Diagnostic dilemma occurs especially in tiny samples, when only small cell or chondroid part of the lesion is available. Final histopathological report in such cases may be delimited to differential circle containing few possible entities. MCS is a rare tumor which should be considered especially in young adult patients with tumors affecting face bones, ribs, vertebrae, pelvis, femur, humerus, and soft periosteal tissue, especially meninges. 3 4 5

Genetic nature of MCS is poorly understood due to its rarity. Most regular abnormalities concern chromosome 8 with −8, +8 and structural aberrations in 8q including *HEY1-NCOA2* fusion. Fusion of these two neighboring genes in most cases is due to deletion of small interstitial region of DNA and probably leads to activation of Notch pathway. The next recently described translocation in MCS is t(1;5)(q24;q32) involving genes *IRF2BP2-CDX1* . Gelderblom et al described a possible another chromosomal abnormality in MCS including der (13;21)(q10;q10). 34 Probably, even more molecular aberrations are engaged in pathogenesis of this neoplasm. 35 36

In general, the overall prognosis for patients with a MCS is poor regardless of the site of the tumor's occurrence because of the tendency of the tumor to hematogenous and lymphatic metastases, most frequently to lungs, lymph nodes, and other bones. 4 16 Despite MCSs slow-growing nature, the tendency of local recurrence and their resistance to chemotherapy or radiation therapy is typical. Local recurrences characterized the clinical course preceding disseminated or pulmonary metastases emphasizing the significance of adequately radical local therapy. 4 The treatment for chondrosarcomas is primarily surgical, obtaining wide surgical margins to achieve local eradication. 2 3 In the spinal MCS, en bloc resection is sometimes possible. Even in some intraspinal MCS, when tumor is located intradurally with attachment to the dura, there are some possibilities to resect the tumor with margins. 30 In tumors located intradurally extramedullary, but without attachment to dura mater, wide margins are impossible because of anatomical constraints. Thus, a marginal or intralesional excision only can be achieved. 3 Neoadjuvant radiotherapy and chemotherapy may be beneficial. Although, because of rarity of MCS, especially located intraspinally, there is no general agreement on the clear protocol of radiotherapy and chemotherapy. The postoperative local radiotherapy may reduce local recurrence rates. De Amorim Bernstein et al suggest to treat all patients with MCS with radiation therapy before and/or after surgery (min. dose of 44 Gy; max. dose of 78 Gy). That way the authors achieved 10-year overall survival rate of 79%. 37 Similarly, Kawaguchi et al stated that treatment of MCS with radiation therapy was significantly associated with improved local-recurrence-free survival. 38 In cases of intraspinal location of the tumor radiotherapy subsequent to surgery with a dose of 50 to 59 Gy was performed. 3 6 27 28 29 In the case, we described, patient was subjected to radiotherapy with similar dose of 4,980 Gy. Postoperative systemic chemotherapy may reduce the risk of metastasis. 21 This modality may be especially useful in cases of unresectable or recurrent tumors. Huvos et al suggested tumors with so-called Ewing-like microscopic features respond somewhat better to combination chemotherapy than those with spindle cell and hemangiopericytoma-like areas. 3 4 Researches by Frezza et al showed that chemotherapy administration in patients with localized disease was associated with fewer recurrences. 7 In retrospective study by Cesari et al, disease-free survival in patients between 5 and 10 years after surgical remission of disease was 76% with chemotherapy and 17% without, although there was no statistical difference in overall survival rate at 10 years between the cohort that received chemotherapy versus no chemotherapy (31 versus 19%). 39 Similar conclusions was described in the series by Dantonello et al. 40 Chemotherapy is based on anthracycline and alkylating agents. In accordance with consensual view, doxorubicin and ifosfamide or cisplatin are recommended. 7 26 34 38

Although concomitant positive role of radiotherapy and chemiotherapy seems to be proven, especially in the unresectable tumors, 5 7 14 there are no convincing recommendation to adjuvant treatment of the patients in the situation of multiple regrowths of the tumor, when there were already performed radiotherapy with critical dose for the neural structures after first recurrence and/or chemotherapy.

Different authors reported variable prognosis in MCS: 10-year survival rates in the literature vary from 21 to 67%. 40 Nakashima et al demonstrated 5- and 10-year survival rates of 54.6 and 27.3%, respectively. 5 De Amorim Bernstein et al reported more favorable 10-year overall survival which was 79%. 37 Local recurrence or distant metastases may appear even many years after the initial treatment. 6 14 Frezza et al reported the appearance of the first lung metastases 20 years after surgery. 7 Nakashima et al reported the first metastasis even 22 years after primary treatment. 5 Some authors have suggested that intraspinal MCS with dural attachment appears to have a more favorable prognosis in comparison with those at other locations. This may be because spinal cord compression by small tumors leads to early diagnosis and early surgical intervention. 3 16 41 42 43 44 Clinical course of MCS is protracted and relentless. It is probably due to low proliferation index of this neoplasm—in our patient stable near 2%. Few patients die within months due to dissemination, and others live for many years until metastatic dissemination. This malignant neoplasm makes long-term follow-up mandatory. 7 45 46 It is very important to choose appropriate therapeutic methods to achieve optimal disease control with a good quality of life of these usually young patients.

Described patient remains in a good neurological condition, despite four surgical resections of the spinal tumors. Undoubtedly, one of the reasons is operation technique with osteoplastic laminotomy. Reconstruction of the laminar roof restores the anatomical barrier which limits the growth of the scar tissue into the spinal canal. When the epidural scar formation is absent in the proximity of the nervous structures, the risk of the damaging thecal sac or nerve roots significantly decreased, especially in case of the surgical treatment of the local recurrence. The described case is enriching the discussion on comparing the usefulness of laminotomy and laminectomy in surgical treatment of the tumors of the spinal canal region and gives an important argument for using reconstructive techniques. The case described in the work of MCS with 14-year-long follow-up and three recurrences was successfully treated surgically and conservatively.

## Conclusion

The treatment of MCS should be primarily surgical, subsequently supported by radiotherapy and chemotherapy. When the tumor is located intradurally, surgical technique of laminotomy is recommended. It allows to restore anatomical bone barrier which, in turn, is decreasing risk of the inadvertent damaging of the neural structures during presumable surgery of the tumor's recurrence. Because of characteristic for MCS tendency to local regrowth and possible late metastases, long-term monitoring of the patients is essential.
